# P02.119. Cognitively-Based Compassion Training reduces peripheral inflammation in adolescents in foster care with high rates of early life adversity

**DOI:** 10.1186/1472-6882-12-S1-P175

**Published:** 2012-06-12

**Authors:** T Pace, L Negi, B Donaldson-Lavelle, B Ozawa-de Silva, S Reddy, S Cole, L Craighead, C Raison

**Affiliations:** 1 Emory University, Atlanta, USA; 2 Research Design Associates, Inc., Yorktown Heights, USA; 3 University of Arizona College of Medicine, Department of Psychiatry, Tucson, USA

## Purpose

Children exposed to early life adversity (ELA) demonstrate elevated circulating concentrations of health-relevant inflammatory biomarkers which persist into adulthood. Increased inflammation in individuals with ELA is believed to contribute to the increased risk for medical and psychiatric illnesses observed in these individuals. The objective of this study was to determine whether Cognitively-Based Compassion Training (CBCT) reduces salivary concentrations of C-reactive protein (CRP) in adolescents with high rates of ELA, and to evaluate the relationship between CBCT practice time and changes in CRP. CBCT is a meditation-based program designed to enhance compassion for self and others and to promote prosocial behavior. Based on prior findings, it was hypothesized that practice time during the study would be more strongly associated with reductions in CRP than would group assignment.

## Methods

Seventy-one adolescents between the ages of 13 and 17 (31 females) in the Georgia Foster Care system were randomized to either six weeks of CBCT or a wait-list control condition. Saliva was obtained upon awakening prior to randomization and again 6 weeks later, as were self-report measures of depression and anxiety. Saliva was assayed for CRP using a high-sensitivity ELISA (Salimetrics, State College, PA). Trauma/neglect history was obtained from state records. Participants completed practice time diaries as a means of assessing amount of engagement with the CBCT program.

## Results

No between group differences were observed in salivary CRP concentrations, self-reported depression or anxiety symptoms. Within the group randomized to CBCT, increased practice time was associated with reduced CRP from baseline to the six week assessment.

## Conclusion

Engagement with CBCT positively impacts an inflammatory biomarker relevant to health in adolescents at high risk for poor adult health and social functioning as a result of significant early life adversity, including placement in foster care.

